# Laparoscopic Excision of an Infected Urachal Sinus in a Teenager: A Rare Case Report With Histological Highlights

**DOI:** 10.7759/cureus.29371

**Published:** 2022-09-20

**Authors:** Jojo James, Nitya Rao, Farah Rana

**Affiliations:** 1 Surgery, Tata Main Hospital, Jamshedpur, IND; 2 General Surgery, Tata Main Hospital, Jamshedpur, IND; 3 Pathology, Tata Main Hospital, Jamshedpur, IND

**Keywords:** umbilical discharge, laparoscopic technique, urachal sinus, teenager, infected urachal cyst

## Abstract

During the development of the coelomic cavity, there is accessible communication between the urinary bladder and the umbilical wall through the urachus. Persistence of this tract results in urachal pathologies with variable symptoms. We present a case of an infected urachal cyst presenting as an umbilical mass with clear discharge in a 19-year-old male successfully managed laparoscopically.

## Introduction

Urachal anomalies are developmental defects that arise during the formation of the urinary bladder and if infected may cause complications. These may present at various ages. Adults may present with a variety of clinical features depending on the subtype of the anomaly. These cases benefit from an early diagnosis, however, achieving the same can be a challenge due to the rarity of presentation and non-specific symptoms. These conditions are best managed by minimally invasive surgery. Our report highlights a case of urachal sinus and how it was managed with excellent results.

## Case presentation

A 19-year-old patient came to our outpatient department with a history of excruciating pain in the umbilicus region; later, the umbilicus was swollen and leaked a yellow-colored clear discharge without any ammoniacal smell. The discharge was continuous, not associated with pain, and had no periodicity. The patient also had three episodes of fever associated with discharge, followed by subsidence of both. The patient was evaluated elsewhere and referred with an ultrasound abdomen report suggestive of an umbilical granuloma.

The patient was evaluated in our department. His vitals were stable, and he was afebrile at the presentation. On examination, there was 1cm x 1cm umbilical swelling that was tender on deep palpation, non-reducible, and without any discharge or cough impulse. The patient had a soft abdomen without generalized or localized tenderness, guarding, or rigidity.

**Figure 1:**
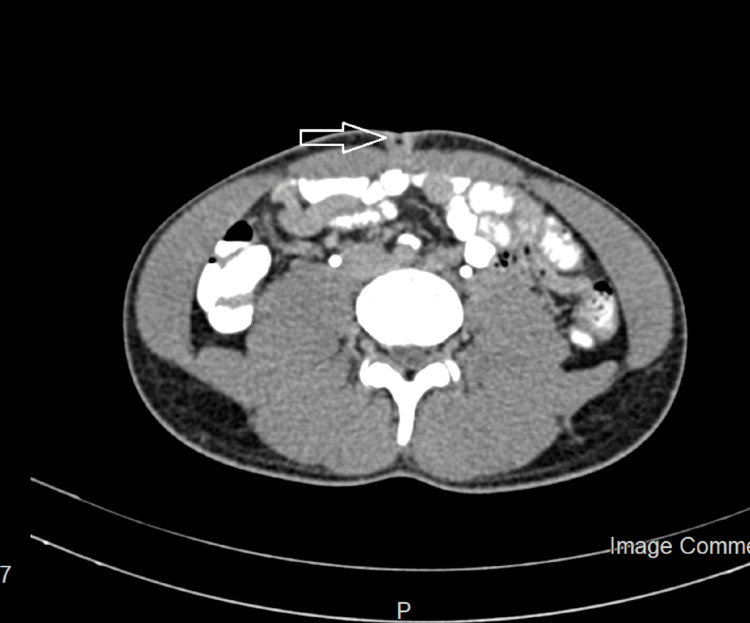
The CT Scan showing urachal sinus at the umbilicus

On routine abdomen ultrasound, a heterogeneously hypoechoic lesion measuring 5cm x 4cm was noted in the umbilical region, with features suggestive of umbilical granuloma seen without any intraperitoneal extension of lesion or collection. A contrast-enhanced computed tomography (CECT) of the abdomen was done, subsequently showing an umbilical soft tissue nodule with a connection to the preperitoneal space with no underlying collection suggestive of an infected urachal cyst (Figure 1).

**Figure 2:**
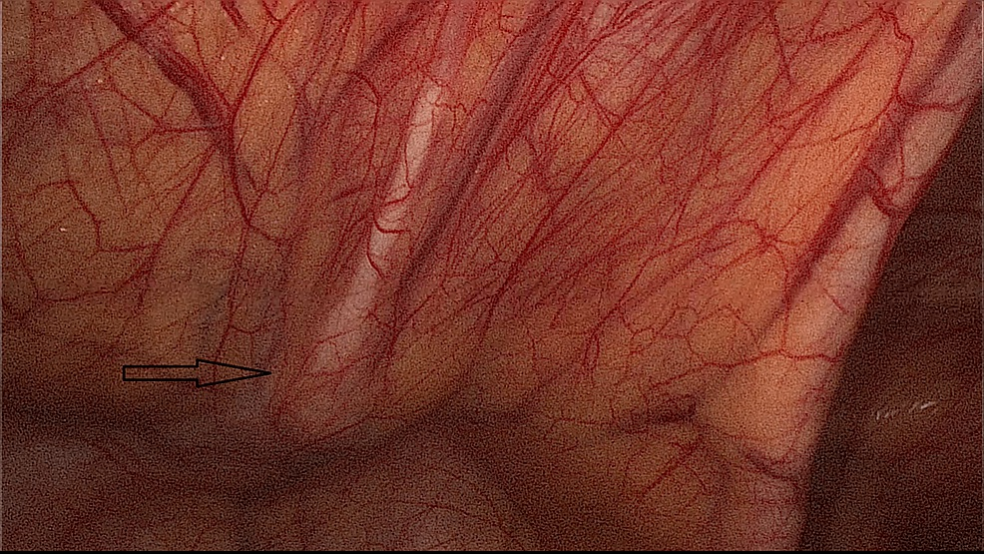
Urachal cyst near bladder with sinus tract

The patient was prepared for a diagnostic laparoscopy and was assessed as fit for anesthesia as per the American Society of Anaesthesiologists (ASA grade 1). Intraoperatively an infected urachal cyst was seen arising from the dome of the urinary bladder of size 5cm x 4cm with a persistent fistulous tract opening into the umbilicus (Figure 2).

**Figure 3:**
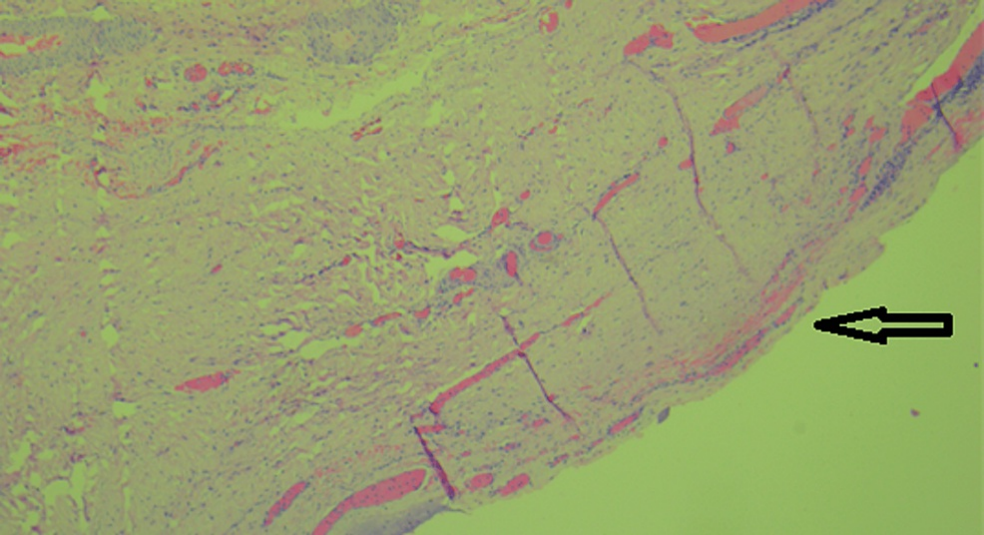
Hematoxylin and eosin stain showing fibroconnective vascular tissues (40×)

**Figure 4:**
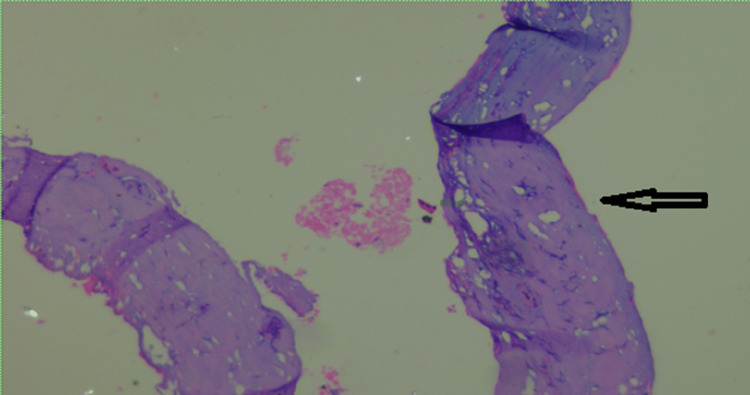
Hematoxylin and eosin stain showing atrophic flattened epithelium lining of the cyst (40×)

**Figure 5:**
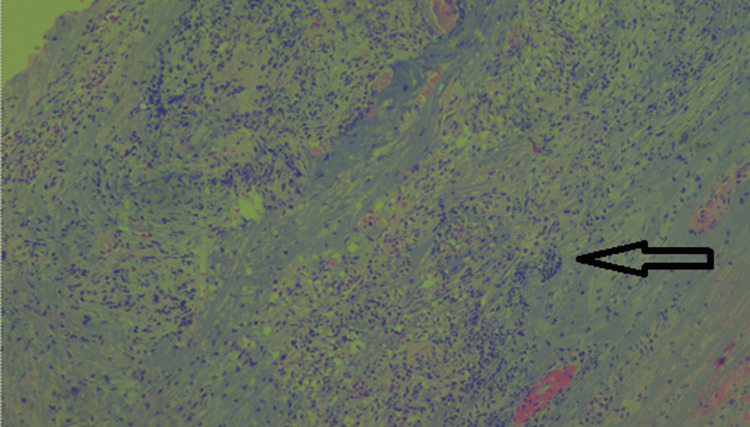
Hematoxylin and eosin stain showing foci of lymphoid infiltrate in the cyst wall (200×)

**Figure 6:**
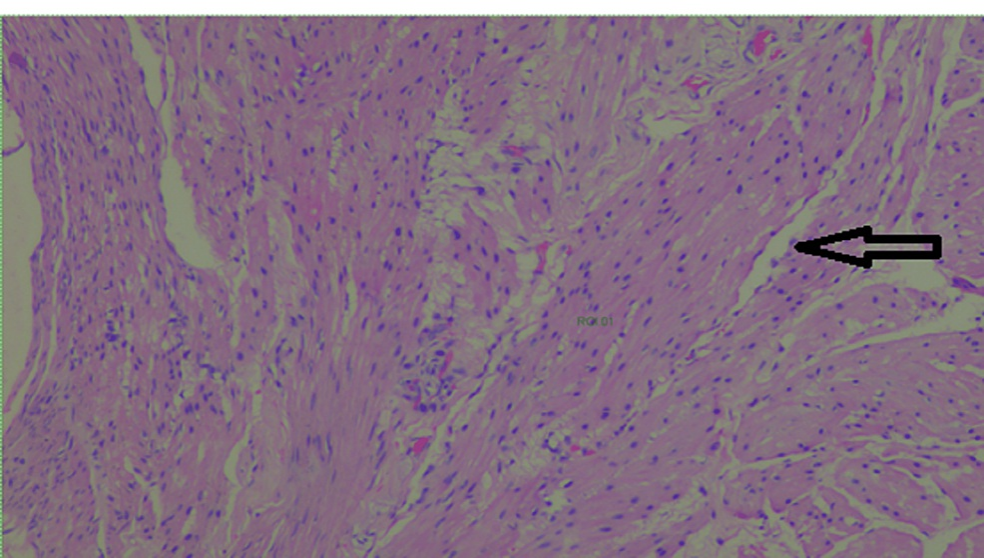
Hematoxylin and eosin stain showing smooth muscle bundles in thickened area cyst sections (400×)

Laparoscopic excision of the urachal cyst with excision of the entire fistulous tract till the umbilicus was done. The integrity of the urinary bladder mucosa was checked intraoperatively after filling the bladder with normal saline, and no leak was detected. A thin drain was placed in the pelvic cavity whose output was recorded daily: 180cc on the first postoperative day gradually reduced to 50cc on the third post-operative day when the drain was removed. On discharge from the hospital, the patient was asymptomatic. He was followed up for four months and was back to playing competitive football tournaments. Histopathological evaluation of our resected specimen with hematoxylin and eosin (H&E) stain showed a partially opened cystic structure measuring 4.6x4.2x1.5 cm. The outer surface was covered with fatty tissues. The cut section showed an irregular granular surfaced cyst wall lining. The wall was variably thickened and firm. No growth was identified. Multiple representative sections were examined, showing fibro adipose predominantly, fibromuscular tissues with focal surface flattened epithelium and mostly atrophic denuded lining. Few lymphoid aggregates and several engorged dilated and some thrombosed thin-walled vessels were seen within the cyst wall. There was no evidence of enteric lining epithelium or malignancy. All findings were compatible with a benign urachal cyst (Figures 3-6).

## Discussion

An embryonic connection between the urinary bladder dome and umbilicus characterizes a urachus. The urachus is a vestigial remnant of the cloaca, which is the cephalic extension of the urogenital sinus, a precursor of the fetal bladder and the allantois which is a derivative of the yolk sac. In urachal anomalies, this tract is partially obliterated and lies in the extraperitoneal plane beneath the umbilicus [1]. This isolated cyst may be identified by ultrasound [2]. This cyst is an inflammatory mass inferior to the umbilicus [3]. Delayed treatment may have severe consequences like sepsis, fistula formation, and rupture, leading to peritonitis. The urachal spectrum of pathologies can present as any one of the following five types: 1) patent urachus, in which the entire tubular structure fails to close; 2) urachal cyst, in which both ends of the canal close, leaving an open central portion; 3) urachal sinus, which drains proximally into the umbilicus; 4) vesicourachal diverticulum, where the distal communication to the bladder persists; and 5) alternating sinus, which can drain to either bladder or umbilicus [4]. Congenital urachal anomalies are infrequent, with various subtypes presenting at varying age groups with men being affected twice more than women, amongst which urachal cyst is the most common presentation in the adult age group [5]. Presentation is atypical; therefore, a high suspicion index helps reach a diagnosis. A triad of lower midline mass, umbilical discharge, and sepsis are suggestive, although MRI confirms the diagnosis and defines the surrounding anatomical relationship. Complete excision is essential because malignant degeneration of the remnant is possible [6]. Histologically, a urachal cyst is usually seen as tubules separated by fibrous cords without marked tissue desmoplasia. The cyst comprises three layers: stratified columnar or urothelial lining epithelium, connective tissues, and outer smooth muscle. Omphalomesenteric duct remnants show the enteric mucosa rather than urothelial or a flat, attenuated epithelium [7,8].

Compared to the article by Sreepadma et al., our case is unique. We have clearly demonstrated that the laparoscopy technique of excision of the urachal cyst has benefited the patient by providing excellent results in terms of surgery with early rehabilitation [9]. The varied presentation of a urachal sinus is a diagnostic challenge hence a high index of suspicion needs to be maintained to achieve an accurate diagnosis and this is where laparoscopy is a useful adjunct in confirming the diagnosis too.

## Conclusions

This case was an unusual presentation of a urachal sinus in this age group and was diagnosed appropriately because of a high index of suspicion. The in-depth histological slides confirm that most of these urachal cysts become symptomatic only after they get infected. Excising the complete urachal sinus tract extending from the urinary bladder to the umbilicus is the right surgical principle to achieve a complete cure and prevent any recurrences. Laparoscopy has not only helped this patient by supporting the diagnosis but it has also helped in the early rehabilitation of this patient. Understanding the anatomy and imaging features of urachal remnant diseases, along with the typical locations and distributions of these diseases, is essential for correct diagnosis and proper management.
